# Rarefied gas flow in functionalized microchannels

**DOI:** 10.1038/s41598-024-59027-1

**Published:** 2024-04-12

**Authors:** Simon Kunze, Pierre Perrier, Rodion Groll, Benjamin Besser, Stylianos Varoutis, Andreas Lüttge, Irina Graur, Jorg Thöming

**Affiliations:** 1 Chemical Process Engineering CVT, University of Bremen, Leobener Str. 6, 28359 Bremen, Germany; 2 IUSTI UMR 7343, CNRS, Aix Marseille Université, 13453 Marseille, France; 3 Center of Applied Space Technology and Microgravity, University of Bremen, Am Fallturm 2, 28359 Bremen, Germany; 4 Karlsruhe Institute of Technology KIT, Hermann-von-Helmholtz-Platz 1, 76344 Eggenstein-Leopoldshafen, Germany; 5 Center for Marine Environmental Sciences MARUM, University of Bremen, Klagenfurter Str. 2-4, 28359 Bremen, Germany; 6 MAPEX Center for Materials and Processes, University of Bremen, Postfach 330 440, 28334 Bremen, Germany

**Keywords:** Gas separation membranes, Mesopores, Carbon dioxide, Knudsen number, S-model, BGK equations, TMAC, Engineering, Physics

## Abstract

The interaction of rarefied gases with functionalized surfaces is of great importance in technical applications such as gas separation membranes and catalysis. To investigate the influence of functionalization and rarefaction on gas flow rate in a defined geometry, pressure-driven gas flow experiments with helium and carbon dioxide through plain and alkyl-functionalized microchannels are performed. The experiments cover Knudsen numbers from 0.01 to 200 and therefore the slip flow regime up to free molecular flow. To minimize the experimental uncertainty which is prevalent in micro flow experiments, a methodology is developed to make optimal use of the measurement data. The results are compared to an analysis-based hydraulic closure model (ACM) predicting rarefied gas flow in straight channels and to numerical solutions of the linearized S-model and BGK kinetic equations. The experimental data shows that if there is a difference between plain and functionalized channels, it is likely obscured by experimental uncertainty. This stands in contrast to previous measurements in smaller geometries and demonstrates that the surface-to-volume ratio of 0.4 $\upmu$m^-1^ seems to be too small for the functionalization to have a strong influence and highlights the importance of geometric scale for surface effects. These results also shed light on the molecular reflection characteristics described by the TMAC.

## Introduction

When the mean free path of gas molecules approaches the smallest size of the surrounding geometry, i.e. pore or channel diameter, the gas is considered to be in a rarefied state. This state is of relevance for many scientific and technical applications, for example gas separation membranes^1^, catalysis^2^, vacuum^3^ and space technologies^4^. Gas behaves differently in a rarefied state and cannot be described by the continuum approach, therefore more general kinetic methods need to be used^5^. In addition, under rarefied conditions the number of collisions between gas molecules and surface is increased compared to the intermolecular collisions of the gas which strongly influences the gas flow. This effect is utilized to increase selectivity in membrane applications. A surface can be functionalized by attaching molecules with specific functional groups which selectively interact with the gas molecules.

However, previous experimental studies have shown that applying such surface functionalization to $\gamma$-alumina ceramic membranes and mesoporous glass membranes significantly reduces the gas flow^6–8^. It was shown that length and density of the functional molecules are the determining factors which impact the flow reduction^9,10^. The chemical composition of the functional group itself does not influence the gas flow.

To investigate this behavior in a larger and, compared to porous media, well-defined geometry, pressure-driven gas flow experiments in straight rectangular microchannels are performed. Hexadecyltrimethoxysilane (HDTMS), a silane molecule with a C_16_ chain as functional group, is deposited onto the channel surfaces by chemical vapor deposition and its influence on the gas flow is investigated.

The experimental results are analyzed for differences in mass flow rate between plain and functionalized channels and the data is compared to numerical solutions of the linearized S-model kinetic equation^11^, the linearized Bhatnagar-Gross-Krook (BGK) kinetic equation^12^, and to an analysis-based hydraulic closure model (ACM) proposed in^13^.

## Methods

The mass flow measurements are performed with the microchannels described below in a bench-scale setup at the IUSTI laboratory in Marseille, France and in the TRANSFLOW facility at the KIT laboratory in Karlsruhe, Germany. The microchannels are prepared at CVT and MARUM in Bremen, Germany.

### Microchannel characteristics

#### Geometry and socket

The microchannels are manufactured by wet etching on silicon wafers, with the channels aligned parallel to the wafer surface. The etching depth corresponds to the channel height and the width is fixed by the masking of the wafer. After anodic bonding with borosilicate glass to close the channels, the length is set by cutting the wafer at specific points.

Before the bonding process, the width and height of the channels are measured via vertical scanning interferometry (VSI). VSI is an optical, non-destructive method (e.g.,^14,15^) that uses white light in the present application. The vertical resolution of the instrument is typically about 1 nanometer, the lateral resolution varies depending on the (mirau) objectives used. The length of the channels is measured using a caliper gauge. Two different channel geometries are used. For the mass flow measurements at the IUSTI laboratory, a stack of 100 parallel channels is manufactured, of which 99 channels are closed by using epoxy glue (*UHU Plus Endfest*) to perform single-channel measurements. For the KIT laboratory, 20 channels in parallel are used for increasing the mass flow to meet the facility’s requirements. The geometries and dimensions of the channels are given in Table 1.

**Table 1:** Channel characteristics.

Channel size	Laboratory	Height h ($\upmu$m)	Width w ($\upmu$m)	Length L (mm)	Parallel channels
Small	IUSTI	5.21 ± 0.1	145.22 ± 0.21	12.07 ± 0.06	1
Large	KIT	48.2 ± 0.3	1469 ± 6	12.56 ± 0.06	20

**Figure 1:**
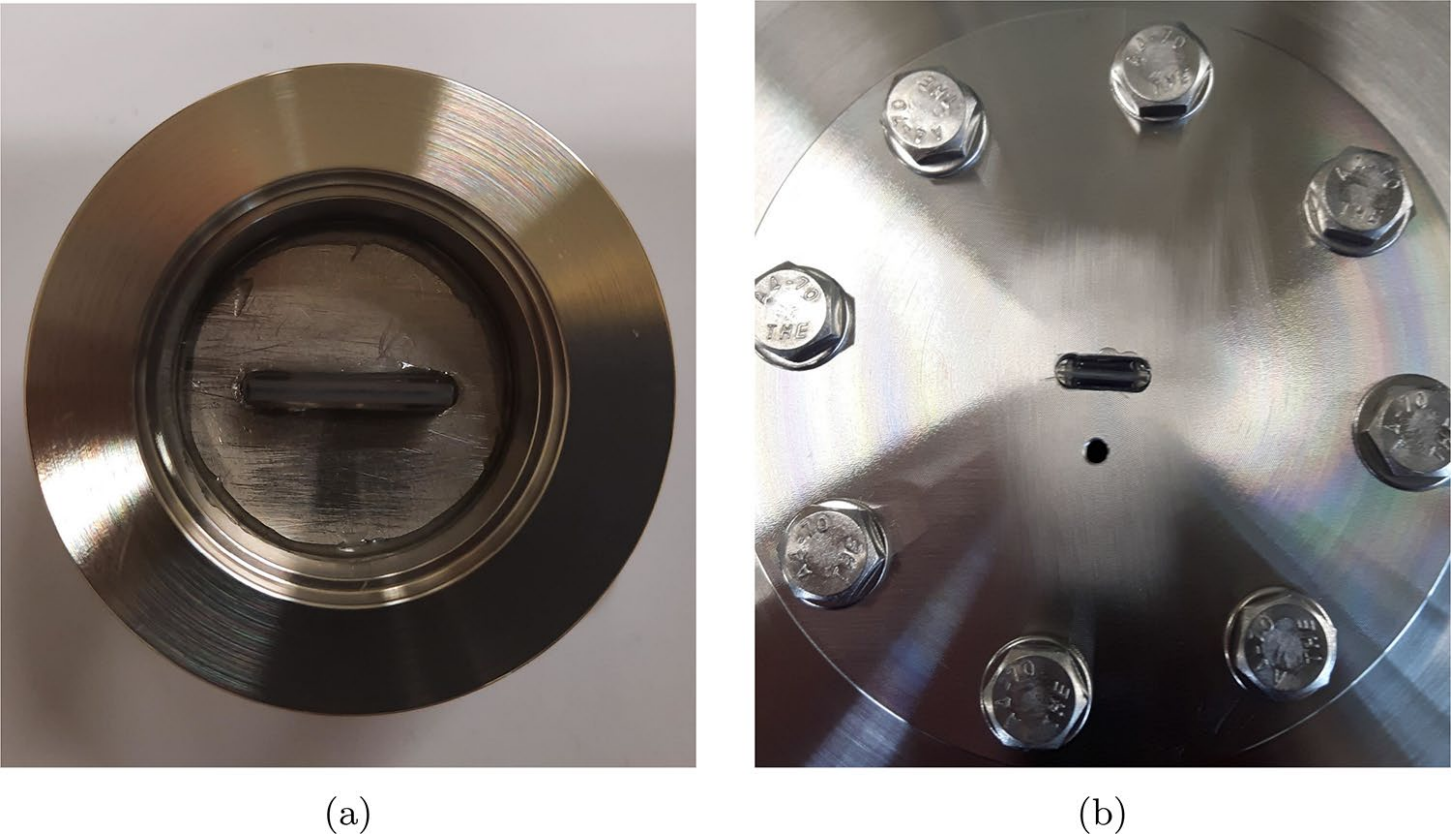
Channel sockets for **(a)** the IUSTI experimental facility and **(b)** the TRANSFLOW experimental facility.

For integrating the microchannels into the measurement setups, they are glued with the same epoxy into a socket with KF (ISO quick release) flanges on each side for the experiments at the IUSTI, see Fig. 1a, and into a CF (ConFlat, cooper-sealed) flange for the experiments at the KIT, see Fig. 1b.

#### Functionalization

**Figure 2:**
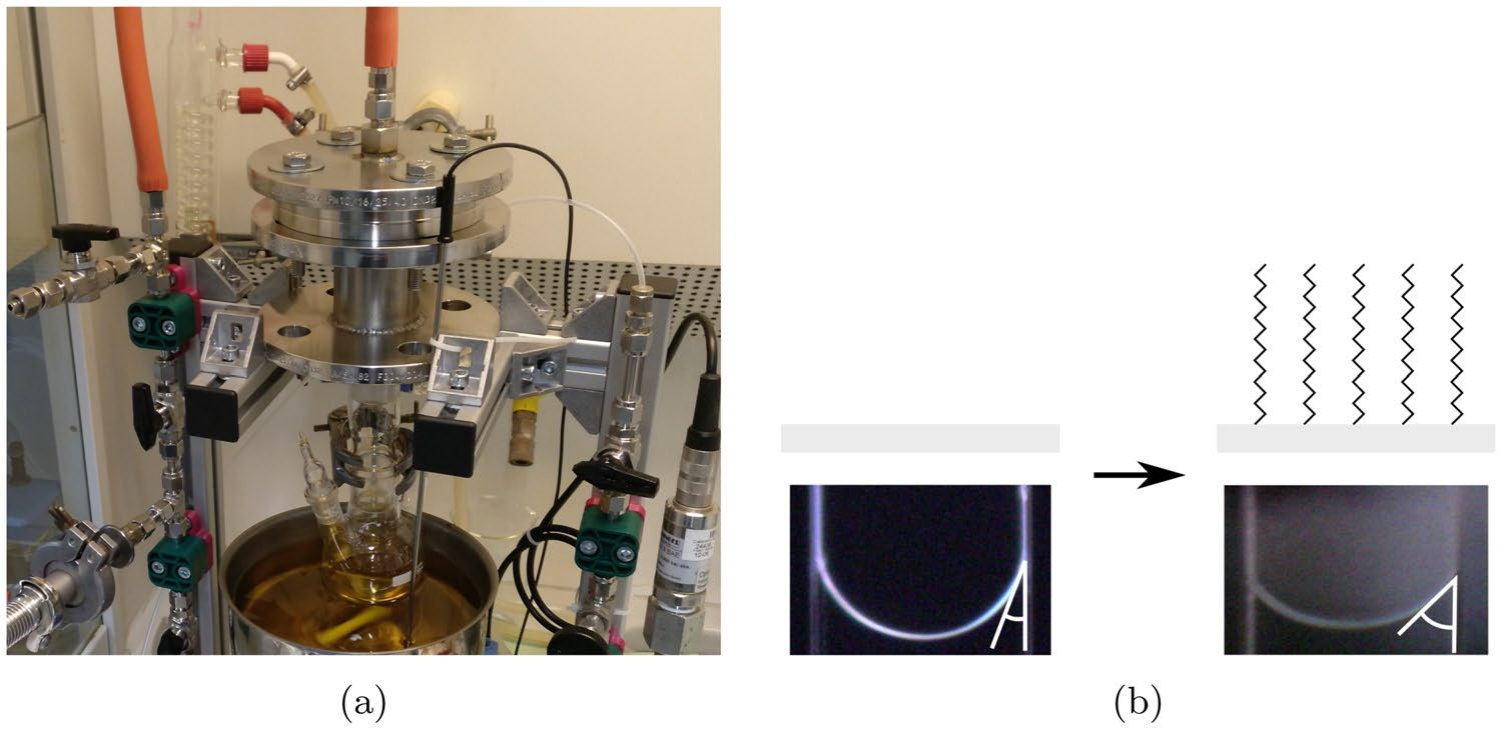
**(a)** The functionalization setup. **(b)** Evaluation of functionalization by contact angle measurement (white) with water. Left: the plain channel. Right: the channel with HDTMS functionalization.

The functionalization of the microchannels is performed by an in-house apparatus for chemical vapor deposition built at the University of Bremen, see Fig. 2a. Only the small channels used at the IUSTI are functionalized, because in those the largest influence is expected. The channel socket attaches to the apparatus by KF flanges. The system is evacuated and flushed with nitrogen. After that, HDTMS is injected into the three-neck round-bottom flask with nitrogen excess pressure. While heating the flask in an oil bath to 150 ^∘^C, the flask is being evacuated until the goal temperature is reached and condensate on the flask is visible. Then, vacuum is only pulled from the other side of the channel, creating a pressure difference along the channel and forcing the silane to flow through it. This process runs for 62 hours. The system is then flooded with air, and the channels are extracted.

To evaluate the result of functionalization, the contact angle of water and air inside the channel is measured, with and without functionalization, see Fig. 2b. For this, the microchannels are observed with an incident light microscope while water is inserted into the channel using a pipette. The results of the contact angle measurements (16 ± 1.3^∘^ for plain, 36.3 ± 2.2^∘^ for functionalized surface) show a clear difference and therefore a successful functionalization. The analysis for the contact angle is performed by using ImageJ^16^ with the contact angle plugin.

### Mass flow rate measurement facilities

**Figure 3:**
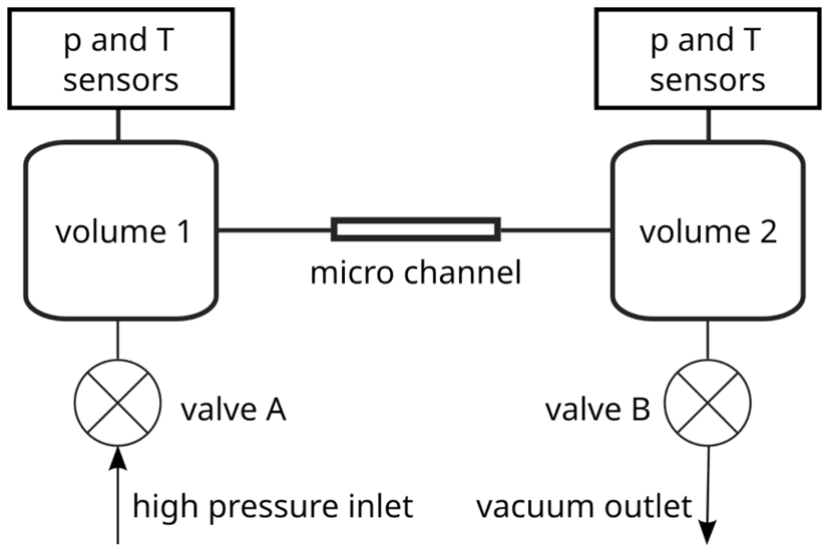
Schematic view of the experimental setups. During an experiment, valve B is closed for the IUSTI setup and open for the TRANSFLOW setup. Valve A is closed in both setups.

In both facilities, the constant volume method is used. This means that pressure changes in the upstream (and optionally downstream) reservoir are constantly measured, while the mass flow rate is deduced from the slope of the pressure evolution over time. The setups are schematically depicted in Fig. 3.

**Figure 4:**
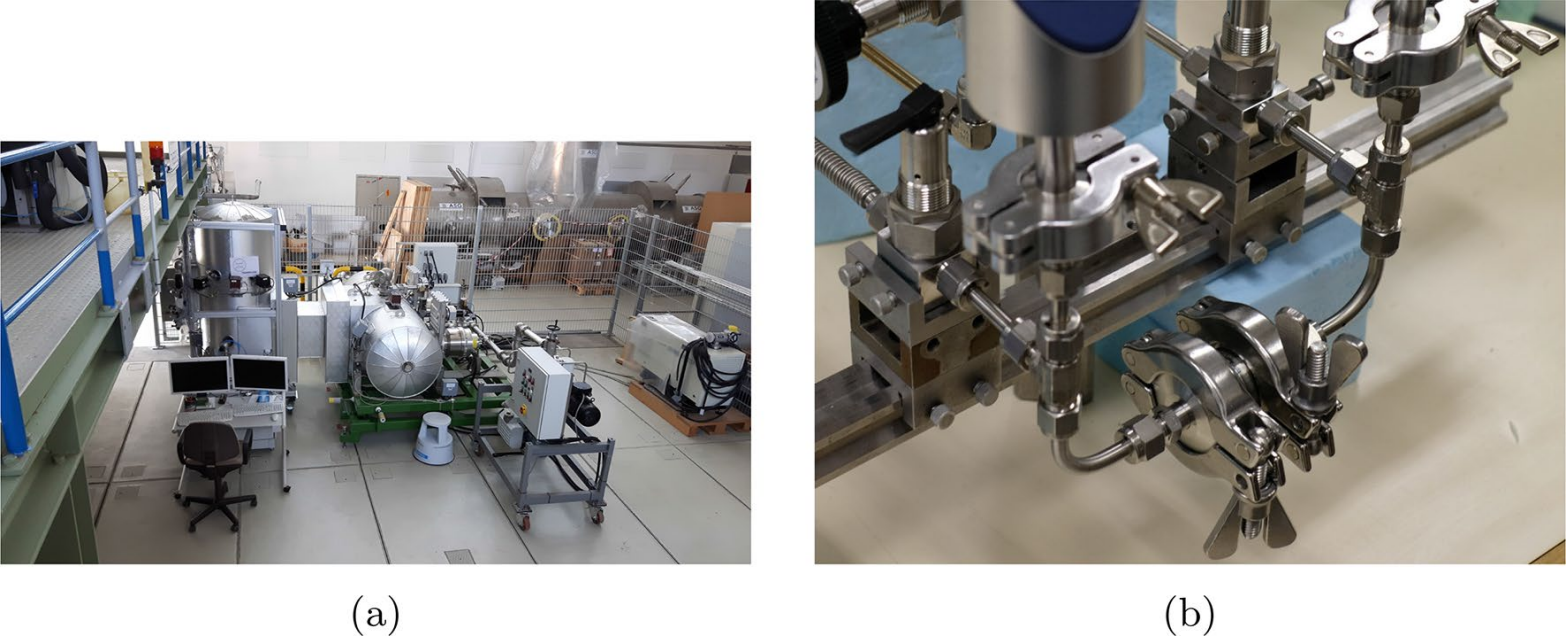
**(a)** Experimental facility TRANSFLOW. **(b)** Experimental facility at the IUSTI laboratory.

In the TRANSFLOW experimental facility, see Fig. 4a, the mass flow is measured by the constant volume method using only the upstream volume. The volume of the upstream reservoir including adapter flange is $V_1$ = (0.606 ± 0.012) $m^3$. Both reservoirs consist of 316LN stainless steel. All connected flanges are high vacuum CF flanges with copper sealing or Swagelok®connections. In the downstream reservoir, two turbomolecular pumps MAG W 2800 by Oerlikon-Leybold are attached via a VAT UHV gate valve. The volume of the downstream reservoir is $V_2$ = 1.2$m^3$ and is assumed quite large compared to the volume of the corresponding test channel. The whole facility can be heated with 6 heating circuits on the upstream, 7 on the downstream reservoir and one on the test channel. The temperatures can be adjusted within 1 degree.

To measure the pressure in both reservoirs, capacitance diaphragm gauges (CDG) by MKS, type Baratron®690 HA, and one hot cathode gauge by Granville-Philips, type Stable Ion®, are installed on respectively. The CDGs provide a maximum measurement range of 133,300 Pa, 1333 Pa and 133.3 Pa at the dosing dome or 13.33 Pa at the pumping dome, respectively. Further details about the TRANSFLOW facility can be found in^17^.

For the facility at the IUSTI laboratory, the constant volume method based on pressure measurements in both upstream and downstream volumes is used to determine the mass flow rate through a microchannel fixed between two volumes, see Fig. 4b. A detailed description of the setup is given in^18^. The facility was slightly modified to work with the channel socket described in “Microchannel characteristics”. To minimize the experimental time needed for a data point, the constant volumes 1 and 2, see Fig. 3, are reduced and they consist merely of the piping. Those volumes are measured by pressure equilibration using a known third volume. The values of $V_1$=(17.6 ± 0.4) cm^3^ and $V_2$=(17.2 ± 0.6) cm^3^ are obtained for the upstream and downstream volumes, respectively. During each experimental run the temperature is measured by K type thermocouples in combination with four-wire Pt100 for cold junction compensation. The temperature is stable within 3.1 K during an experimental run with a maximum length of around 17 h and corresponds to room temperature. With this setup it is possible to measure pressures in the range of 10 to 100,000 Pa with Capacitance diaphragm gauges (CDG). This type of sensor has an uncertainty of 0.2% of reading. Two gases were used, helium and carbon dioxide, provided by Air Liquide (France) with a purity of 99.999%. The available pressure range corresponds to a Knudsen number range of 0.03 to 10 for helium and of 0.01 to 3 for carbon dioxide.

At the TRANSFLOW facility, we performed experiments using plain large channels with helium. At the IUSTI facility, we conducted experiments using small channels with and without functionalization with helium as well as with carbon dioxide.

Leakages could impact the measurements when working under atmospheric pressure. Therefore, for the Transflow facility a helium leak test is performed after each channel installation to exclude any significant leakage. For the IUSTI facility for each channel-gas-gauge combination a leakage measurement is performed which is then used to correct the actual measurement runs, see “ General mass flow rate calculation” section 2.3.

### Method for optimized mass flow rate measurement

#### General mass flow rate calculation

When the pressure in the volumes is changing due to the mass flow through the channel and it does so slowly in relation to the time needed to reach a local equilibrium, we can assume that the system is in a quasi-stationary state. Therefore, under the quasi-stationary state assumption, we can consider the change in the system as a succession of local equilibria. For a single gas and constant volume of a tank $V_i$ the change in mass of a gas $m_i$ inside is given by

1 $$\begin{aligned} \textrm{d}m_i = m_i \left( \frac{\textrm{d}p_i}{p_i} - \frac{\textrm{d}T_i}{T} \right) \end{aligned}$$

where $p_i$ is the pressure and $T_i$ is the temperature in tank *i*. Over an infinitesimal time interval, we can calculate the mass flow rate as

2 $$\begin{aligned} \frac{\textrm{d}m_i}{\textrm{d}t} = \frac{V_i}{R T / M} \frac{\textrm{d}p_i}{\textrm{d}t} - \frac{p_i V_i}{R T^2 / M} \frac{\textrm{d}T}{\textrm{d}t} \end{aligned}$$

where R is the universal gas constant and M the molar mass of the gas. The temperature in the tanks is kept stable and nearly constant in time. Therefore, the change of temperature in time compared to the change in pressure in time is very small, so we can consider the mass flow rate due to change of temperature in time negligible

3 $$\begin{aligned} \frac{V_i}{R T / M} \frac{\textrm{d}p_i}{\textrm{d}t} \gg \frac{p_i V_i}{R T^2 / M} \frac{\textrm{d}T}{\textrm{d}t}, \end{aligned}$$

or equivalently, the relative change of pressure is much greater than the relative change in temperature

4 $$\begin{aligned} \frac{\textrm{d}p_i}{p_i} \gg \frac{\textrm{d}T}{T}. \end{aligned}$$

Then, the mass flow rate, ${\dot{m}}$, is simply

5 $$\begin{aligned} \dot{m_i}(t)= \frac{\textrm{d}m_i}{\textrm{d}t} = \frac{V_i}{R T / M} \frac{\textrm{d}p_i}{\textrm{d}t}. \end{aligned}$$

If both valves A and B (see Fig. 3) are closed, which is the case for the IUSTI facility but not for TRANSFLOW, the mass conservation between volume 1 and 2 results in

6 $$\begin{aligned} {\dot{m}}_{1}(t)=-{\dot{m}}_{2}(t) \end{aligned}$$

using the convention of a negative mass flow for a reduction of mass inside a volume.

To calculate the mass flow rate the pressure variation in a tank over the time is measured. At the IUSTI laboratory, the pressure in both upstream and downstream volumes changes: the higher upstream pressure decreases over time while lower downstream pressure increases, until an equilibrium pressure, $p_{eq}$, is reached in both tanks. At the KIT laboratory, the downstream volume is continuously evacuated, therefore only the upstream pressure change is used for mass flow rate calculation.

These pressure variations in time can be fitted using either a linear function or more generally using an exponential function. To be able to cover a larger experimental range where the mass flow might not be constant, an exponential function is chosen here, as proposed in^19,20^:

7 $$\begin{aligned} p_i(t) = p_{eq} + (p_i^*-p_{eq})e^{-t/\tau _i}, \end{aligned}$$

where $p_{eq}$ is the equilibrium pressure for $t \rightarrow \infty$, $p_i^*$ is the initial pressure in tank *i*, and $\tau _i$ is the pressure relaxation time, which characterizes the speed of pressure rise or drop. The measured pressure variation in time is fitted using Eq. (7) with $\tau$ and $p^*$ as the fitting parameters. $p_{eq}$ is approximated using the initial pressures and the tank volumes. The pressure derivative with respect to time is calculated as

8 $$\begin{aligned} \frac{\textrm{d}p_i}{\textrm{d}t} = -\frac{p_i^*-p_{eq}}{\tau _i} e^{-t/\tau _i} \end{aligned}$$

The mass flow rate is then calculated from

9 $$\begin{aligned} \dot{m_i}(t) = -\frac{V_i}{RT/M}\frac{p_i^*-p_{eq}}{\tau _i}e^{-t/\tau _i}. \end{aligned}$$

which simplifies for $t=0$ to

10 $$\begin{aligned} \dot{m_i} = -\frac{V_i}{RT/M}\frac{p_i^*-p_{eq}}{\tau _i} \end{aligned}$$

to get the mass flow rate at the beginning of the experiment.

The measured mass flow rate is corrected with a previously measured leakage or outgassing mass flow rate by addition or subtraction of this flow rate when the leakage or outgassing is significant. The leakage mass flow rate is determined by setting the pressure equal in both volumes and by monitoring the pressure rise over time. The magnitude of the influence of the leakage correction varies strongly between experimental runs and is quantified in Appendix 5.

Since the mass flow is calculated using the pressure drop in the volumes, the experiments need to run for a certain time to have a significant change in pressure compared to the sensor noise. However, a too long measurement time introduces external influences like temperature fluctuation which can impact the temperature constancy assumption, required for implementing described mass flow rate extraction model, see “General mass flow rate calculation”. Too short measurements in turn can lead to a high uncertainty due to the small amount of data available for a fit.

The mass flow rate measurement uncertainty consists of two parts: one part describing the reliability of the chosen amount of measurement data using *data fragments*, see “Variability of data fragments”, and the other part describing the physical uncertainties of pressure sensors, temperature fluctuations and channel and facility geometries, see “Error propagation”. To find the ideal amount of data, the measurement uncertainty is minimized, see “Minimization of uncertainties”.

#### Variability of data fragments

The *variability of data fragments* methodology differs for the IUSTI and the TRANSFLOW facilities.

In case of the IUSTI facility, an important criterion for a successful run is the match of inlet and outlet mass flows, Eq. (6), which are independently calculated using the respective pressure drops. This criterion is not fulfilled if the signal-to-noise ratio is too low, because that will introduce randomness to the mass flows and they likely will differ. Also, temperature fluctuations would create a mismatch because a temperature rise decreases the calculated mass flow rate in volume 1 and increases it in volume 2.

**Figure 5:**
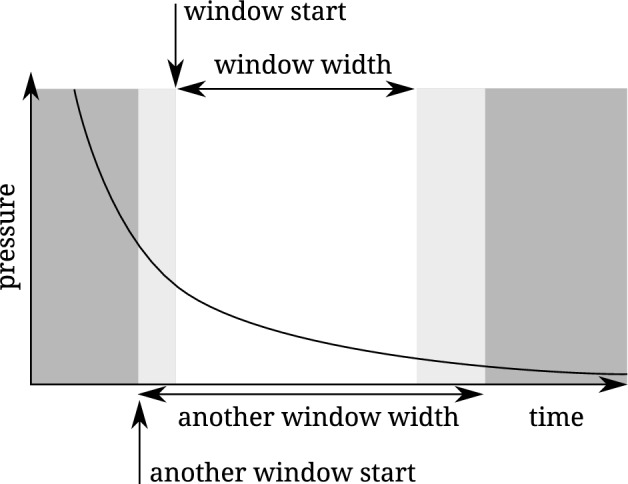
Depiction of two *time windows*. A *time window* is defined by a start and a width. One window consists of the data with white background. Another window consists of the data with white and light grey background: it has an earlier start and a larger width.

**Figure 6:**
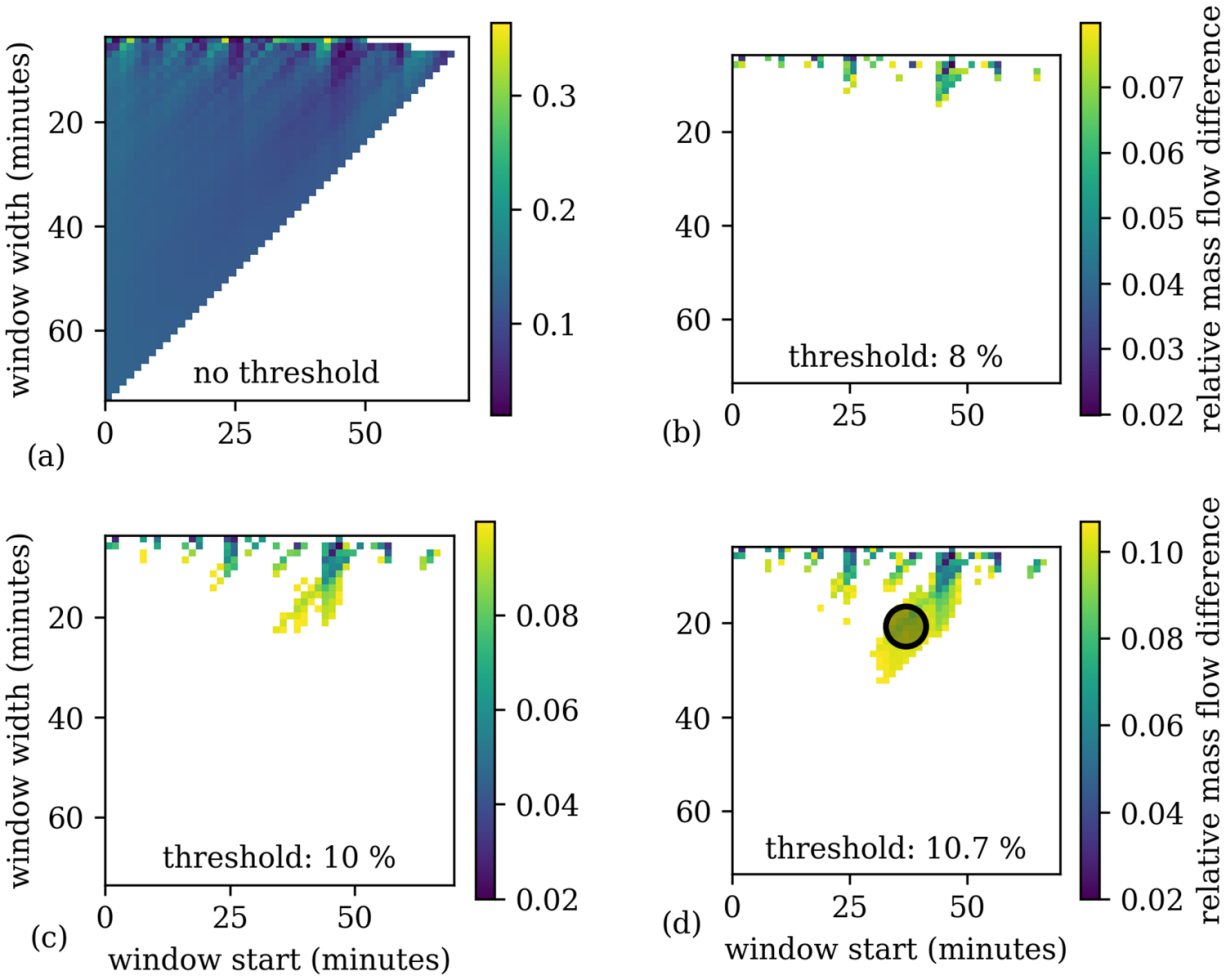
Differences between mass flow rates calculated from upstream and downstream pressure changes. Each square corresponds to a fragment of the data defined by its corresponding window start and window width. The lower right part is empty because the window would end outside the available data. This results in (50x50)/2 = 1250 single evaluations. Some samples at the top are empty because no fit for that data was found. **(a)** The full array without any threshold. **(b)** A threshold of 8 %; only very few fragments fulfill this threshold. No sufficiently large area can be found. **(c)** A threshold of 10%. More fragments fulfill this threshold, but not enough to find a sufficiently large area. **(d)** A threshold of 10.7% results in a structure where a sufficiently large area can be found (marked with a circle). Therefore, the corresponding relative uncertainty of mass flow rate due to the data fragment method is 5.35%.

Accordingly, the issues above can be addressed by looking at the difference of the mass flow rates calculated in volumes 1 and 2. To evaluate the correct experimental time, a single long experimental run is used. Only parts of this run are analyzed to simulate different lengths of experimental runs. These parts are taken at different positions and are of different length, resulting in *time windows* as depicted in Fig. 5. This results in a two-dimensional array of fictive experiments, each having their analyzed mass flow rates and their difference between volumes 1 and 2. An example of a two-dimensional array, visualizing the relative difference between the measured mass flow rates in volumes 1 and 2, is shown in Fig. 6a.

For the runs with small width, very low deviations are right next to very high deviations. This is the result of a low signal-to-noise ratio of little data. Simply choosing the data fragment having the smallest deviation would therefore be a matter of chance and not a reliable choice.

To tackle this problem, a second criterion is imposed: not only does a single fragment need to have a low difference between inlet and outlet, but an *area* of fragments should stay below a certain threshold. This is done by applying a threshold to the array of mass flow differences and searching for an area large enough to host a circle with a radius of 20% of the width of the time window at the circle’s center. This corresponds to a possible shift of the window start by 20% of the window width and a scaling of the window width by 20% while staying within the threshold. To avoid noise problems, a minimum of three time windows should be covered by the radius. To include as much data as possible for the fit, longer runs are prioritized if ambiguity arises.

The threshold is increased until an area fulfilling the 20% criterion is found. That process is depicted in Fig. 6b–d. The Knudsen number and the dimensionless mass flow rate, defined by Eqs. (12) and (11) respectively, are taken from the run in the center of the circle.

In case of TRANSFLOW, there is no difference between mass flow rates of volumes 1 and 2 because only volume 1 is considered, so the methodology is slightly different. Each entry of the array is investigated one by one. For each entry, the difference to all other fragments is calculated. This results in an array of mass flow differences—not between volumes 1 and 2, but between the current and all other data fragments. To this difference a threshold is applied like above and a check for an area fulfilling the 20 % criterion is performed. If no area is found, the next entry of the array is investigated by re-calculating the difference to all other fragments, applying the threshold and checking for an area. If after cycling through all entries no area is found, the threshold is increased and all entries are investigated one by one again until an area is found.

This threshold is then used for an uncertainty which is defined via the population standard deviation^21^, p. 111 as half of the relative difference between inlet and outlet mass flow rates or half of the relative difference between the current window and all other windows within the found area.

Generally, the method using two volumes should be preferred because the successful comparison of two separate mass flow balances is a strong indicator of correct measurements. Using only one volume leaves more room for systematic errors because influences like leakage or adsorption and desorption effects are not directly detectable.

#### Monte Carlo simulations

To quantify the influence of the pressure sensor uncertainties on the calculated pressure drop, a Monte Carlo simulation is performed. The sensor uncertainty is assumed to follow a normal distribution. For each sample, the error is calculated using the measured data and the sensor uncertainty, which is a constant factor for the IUSTI facility and a pressure-dependent factor according to calibration certificates for the TRANSFLOW facility.

This error is added to the measured data and a fit is performed. The pressure drop is calculated using this fit. This process is repeated with many samples until a convergence of the mean of the pressure drops resulting from the samples is reached. The standard deviation of the pressure drop between the samples is the uncertainty of the pressure drop due to the pressure sensor uncertainty.

#### Error propagation

The influence of the uncertainties of pressure drop (calculated with the Monte Carlo method), temperature (calculated using the standard deviation of temperature during an experimental run), facility volumes (see “Mass flow rate measurement facilities”) and channel geometry (calculated using the VSI data, see “Geometry and socket”) on the dimensional and dimensionless mass flow rates is determined using standard error propagation of independent variables. This is implemented using the Python package uncertainties^22^. A similar type of Monte Carlo error propagation analysis has been performed in Ref.^23^ in pressure driven flows to quantify the effect of the pressure uncertainty in the mass flow rate.

#### Minimization of uncertainties

The total uncertainty is the sum of the uncertainty coming from the data fragment analysis, see “Variability of data fragments”, and the uncertainty calculated by error propagation, see “Error propagation”. To minimize the total uncertainty, the best data fragment needs to be determined. This is done by sampling many thresholds for the array of mass flow differences and performing the complete calculation of the total uncertainty for each threshold. Thresholds with no valid 20% area are discarded. The threshold with the lowest total uncertainty is chosen, and the data fragment associated with this threshold is determined as the optimal data fragment. If this total uncertainty is still larger than 20%, that experimental run is discarded. This is by no means a polishing of data, but rather as a tool to identify experimental runs which have issues at the execution level, like excessive adsorption and desorption effects or leakage.

### Analytical and numerical solutions

For comparison with the experimental data, an analysis-based hydraulic closure model (ACM) and numerical simulations are used.

The ACM model^13^ provides a mass flow rate expression and consists of a superposition of slip as well as diffusive flow. The latter is a combination of Fickian self-diffusion and free molecular flow. The molecular mean free path, which is part of the analytical expression for both the slip flow and the diffusive flow, is replaced by a so-called “effective” mean free path to take into account the channel geometry for high Knudsen numbers. This “effective” mean free path of a molecule at near-free molecular flow is defined not by the pressure or number density but by the size of the surrounding geometry. For the molecular diameters of the gases, also influencing the mean free path, the transition molecular diameters as presented in^13^ are chosen, which show good agreement with literature data. The ACM model is used without further modification for this work.

The relaxation type models of the Boltzmann equation collisional term, like the BKG and S-model, are largely used for simulation of the gas flows through channels of different cross-sectional shapes^24,25^. In this work, the linearized versions of both model kinetic equations, BGK and S-model, are solved numerically. This approach allows to simulate the gas flow through channels with a small ratio between the characteristic dimension of the cross-section and the channel length, $h/L\ll 1$ (which is the case for the channels used in these experiments) and any pressure ratio between the tanks, see more details in^11,12^. It is worth nothing that in these simulations the classical definition of equivalent mean free path, Eq. (13), is used as it is also defined in Ref.^25^. However, both models require additional information about the gas-surface interaction in terms of the accommodation coefficient^24^ which is set to 1 for this work. Furthermore, the fact that a polyatomic gas is treated as a monoatomic in the numerical simulations is fully justified by the results of previous studies^24,26^, where it was found that in an isothermal pressure driven flow, the internal degrees of freedom do not influence the mass flow rate through a duct.

## Results and discussion

### Measured mass flow rate

The measured data is shown in a form of dimensionless mass flow rate, *G*, to exclude the influence of geometric scale and temperature, and reduce that of gas species. This is done by dividing the measured mass flow rate, ${\dot{m}}$, by a mass flow rate often called *Knudsen diffusion*^27^, resulting in a non-dimensional mass flow rate of

11 $$\begin{aligned} G = {\dot{m}} \frac{3 \Pi L}{8A^2 \Delta p} \sqrt{\frac{\pi RT}{2M}} \end{aligned}$$

where $\Pi$ is the channel perimeter, *L* is the channel length, *A* is the channel cross-sectional area, $\Delta p$ is the pressure difference between inlet and outlet tanks.

All experimental results are plotted as a function of the Knudsen number which is defined using the microchannel height, the smallest dimension of the rectangular channel cross-section, as

12 $$\begin{aligned} Kn=\frac{\lambda }{h}, \end{aligned}$$

where $\lambda$ is the equivalent molecular mean free path calculated by

13 $$\begin{aligned} \lambda =\frac{\mu (T)}{p_m^*} \sqrt{\frac{\pi RT}{2M}} \end{aligned}$$

where $\mu (T)$ is the temperature-dependent dynamic viscosity interpolated from data available in^28^ and $p_m^*=0.5(p_1^*+p_2^*)$ is the mean pressure between the inlet and outlet tanks, $p_1^*$ and $p_2^*$, respectively, at the beginning of the experiment.

**Figure 7:**
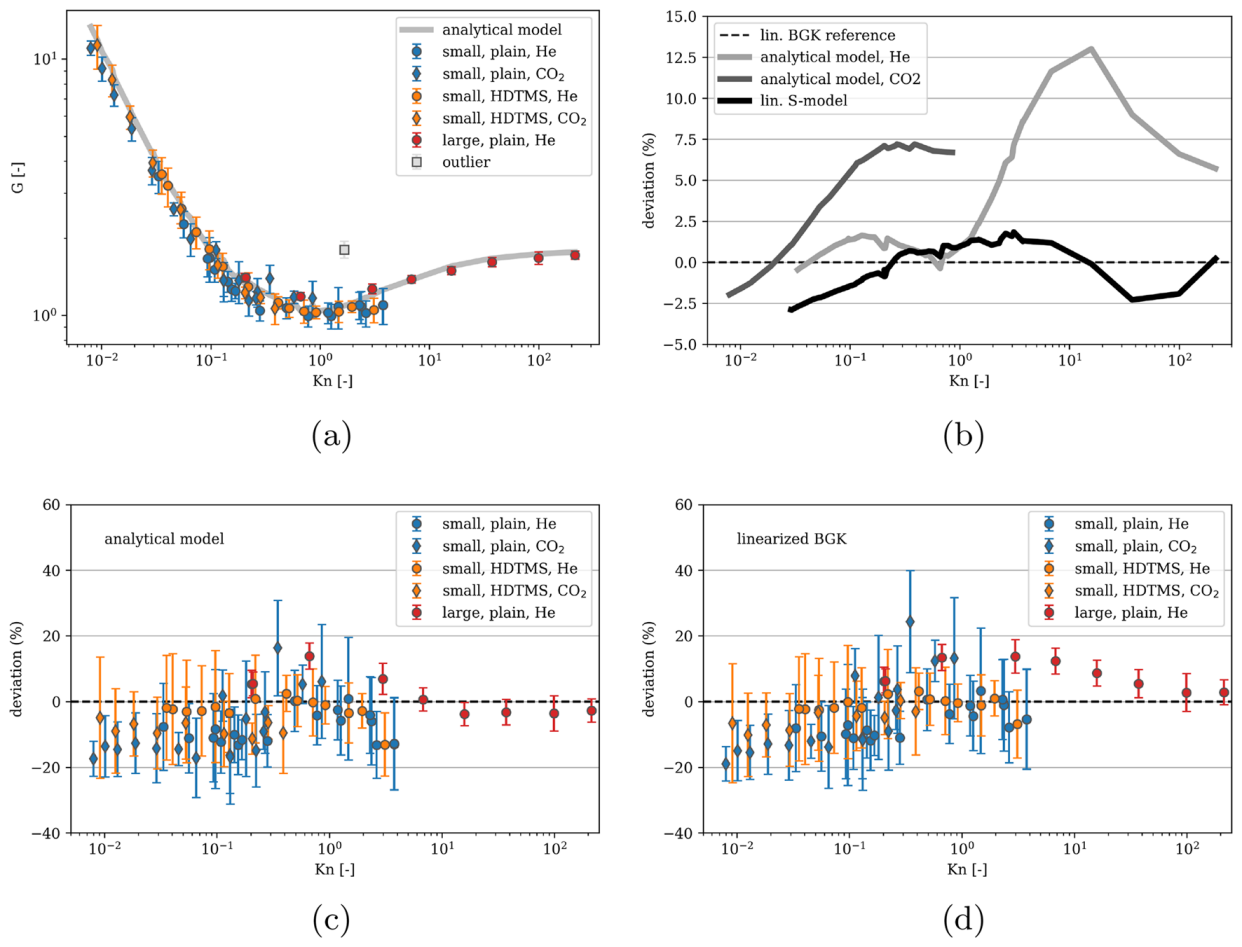
**(a)** Dimensionless mass flow rate obtained experimentally (symbols) and calculated from the analysis-based hydraulic closure model (ACM)^13^ (line). The outlier in grey color is not included in further analysis. **(b)** Deviation of the ACM analytical model and the linearized S-model from the linearized BGK model. **(c)** Comparison of experiments to the ACM analytical model. A negative value corresponds to an experimental value smaller than the analytical one. **(d)** Comparison of experiments to the numerical solution of the linearized BGK equation.

The results of the experiments for both the plain and the HDTMS-functionalized channels with helium and carbon dioxide are shown in Fig. 7a together with the analytical curve. The so-called Knudsen minimum^29^ at around Kn $\approx$ 1 is well visible. The dimensionless mass flow rate converges to a constant value for large Knudsen numbers as expected^30^. One experiment, although fulfilling the criterion of an uncertainty of < 20 %, is considered an outlier and is therefore plotted in grey color and excluded from further discussion.

### Influence of surface functionalization

To assess the influence of surface functionalization, we only use data for the small channels because for the large channels we do not have data with functionalized surfaces. We have four categories of measured data resulting from a combination of two features which are the gas species (helium and carbon dioxide) and type of surface (plain and functionalized). We then compare each of the four combinations of two categories where one feature stays constant to investigate the effect of only one feature, see Table 2. The goal of the comparison is to decide whether there is a difference between the two groups. For this, we perform an independent two-sample t-test with the null hypothesis that both populations have the same distribution. The variances of the populations are not assumed to be equal, resulting in Welch’s t-test^31^.

**Table 2:** Combinations of categories for the t-test.

Surface type	Gas species		
	He	CO_2_	Combination
Plain	He, plain	CO_2_, plain	Plain, He vs. CO_2_
HDTMS	He, HDTMS	CO_2_, HDTMS	HDTMS, He vs. CO_2_
Combination	He, plain vs. HDTMS	CO_2_, plain vs. HDTMS	

The t-test assumes a normal distribution of the data. The experimental data however strongly depends on the Knudsen number. Therefore, we need to transform the dimensionless mass flow *G* to make it as independent on $\textrm{Kn}$ as possible. This can be done by subtracting the most appropriate baseline, which is in our case either the ACM solution or one of the numerical models. To decide which baseline to choose, we investigate the deviation between the baselines. This is shown in Fig. 7b where the deviation of the ACM solution and the linearized S-model from the linearized BGK model is plotted for the whole Knudsen number range. While the linearized S-model barely differs from the linearized BGK model, the ACM solution has a maximum deviation of around 13%. Therefore, both the linearized BGK and the ACM solution are used as a baseline. The t-test is then applied to the *relative difference* between the experimental *G* and the analytical or numerical *G*. The relative difference makes sure that data for small Knudsen numbers is as important as data for large Knudsen numbers. Fig. 7c, d show the relative deviation of all the experimental data from the baselines. For a visualization of the category combinations and their relative deviation from the linearized BGK model results see Fig. 15 in Appendix 6. For a compilation of the deviation of all experimental data from all models see Fig. 16 in Appendix 7.

A normal t-test does not take into account the measurement uncertainty. To improve this, we performed a Monte Carlo sampling of the experimental data and added an offset to each measurement point. This offset is calculated using a normal distribution with the measurement uncertainty as standard deviation. For each sample, we performed the two-sample t-test which results in a p-value and a confidence interval. The confidence interval is calculated using a 95% confidence level. The confidence interval describes the interval for the difference between the means of the population distributions which is zero if there is no effect of surface functionalization.

**Figure 8:**
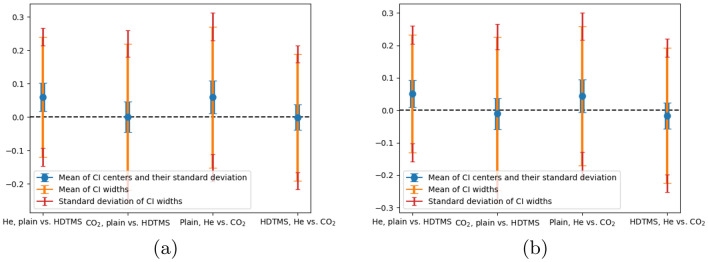
T-test confidence intervals with a 95% confidence level depicting the means and standard deviations of the centers and width of the intervals resulting from the Monte Carlo sampling **(a)** for the analysis-based hydraulic closure model (ACM) baseline and **(b)** for the linearized BGK model baseline.

To analyze the confidence intervals of all samples, two metrics are calculated: the *mean of the centers* of the confidence intervals along with their standard deviation, and the *mean of the confidence interval widths* along with their standard deviation. These metrics are visualized in Fig 8a for the ACM solution baseline and in Fig 8b for the linearized BGK model baseline. For the case of helium in plain and functionalized channels and the case of helium and carbon dioxide in plain channels, the mean of the centers is larger than zero, around 0.05, with the standard deviation just exceeding 0.1. For the other two cases, the mean is very close to zero. While the standard deviation of the centers is comparably small, the width of the intervals are very large and always cover zero. In turn, the standard deviation of the widths is small compared to the actual width, indicating that the width is always pretty large. The most important reason for such large intervals is the high measurement uncertainty which directly influences the interval width during the Monte Carlo sampling. The fact that all these intervals have the same scale suggests that if there is an effect of the functionalization, it is likely obscured by the measurement uncertainty.

It is therefore difficult to state a clear and unambiguous conclusion. The confidence intervals imply that the effect of the functionalization could be anywhere between ± 25 % and zero. Similarly, the same is valid for the difference between helium and carbon dioxide.

Several authors^32,33^ studied the influence of the surface nature on the mass flow rate in the free molecular regime. They found that where a surface is rough it is difficult to distinguish the difference between the surfaces of different nature and the accommodation coefficients will be equal to one whatever the surface nature is. In our study, unfortunately the surface roughness could not be measured, so we cannot explain the absence of the functionnalization effect by this way.

The absence of a pronounced impact of the functionalized surface may be explained by the fact that the influence of gas-surface interaction on the mass flow rate depends not only on the rarefaction level but also on the channel surface-to-volume ratio. This ratio for the channels used in experiments at the IUSTI laboratory is much smaller (0.4 $\upmu$m^-1^) than that of the previous experiments reported in^10^ (300 $\upmu$m^-1^, with a pore diameter of 20 nm). Therefore, the surface to volume ratio for the channels used in the experiments presented here is likely too small to have any influence on the mass flow rate. This can also be explained by imagining two possible trajectories of a molecule in a small and a large channel. For the same travel distance, the number of collisions with the wall is much higher in the small channel compared to the large channel. This corresponds to an increased influence of any existing functionalization molecules on the surface for smaller channels.

### Numerical modeling using the linearized BGK-model kinetic equation

The numerical solution of the BGK kinetic equation has been obtained for the rectangular channel with high-to-width aspect ratio equal to 0.0328, which correspond to the dimension of channels used in KIT experiments. The software developed in^12^ was used.

By comparing the corresponding numerical and experimental results, see Fig. 7d, it can be observed that for monoatomic helium and for the case of the plain as well as the functionalized surface, a good agreement in the range of 0.03 $\le$ Kn $\le$ 110 is obtained, in which the relative error ranges between 1 and 14 %. The magnitude of relative errors is evenly distributed across the Knudsen range.

For the case of the polyatomic gas CO_2_, it is observed that a good agreement in the range of 0.01 $\le$ Kn < 1 is achieved, with the relative error ranging between 2 and 25 %, which is significantly higher than that for monoatomic helium. Large discrepancies are obtained in the case of Kn$\sim$1 and for Kn < 0.1, which are mainly attributed to the high uncertainty of the experimental results.

### Numerical modeling using the linearized S-model kinetic equation

**Figure 16:**
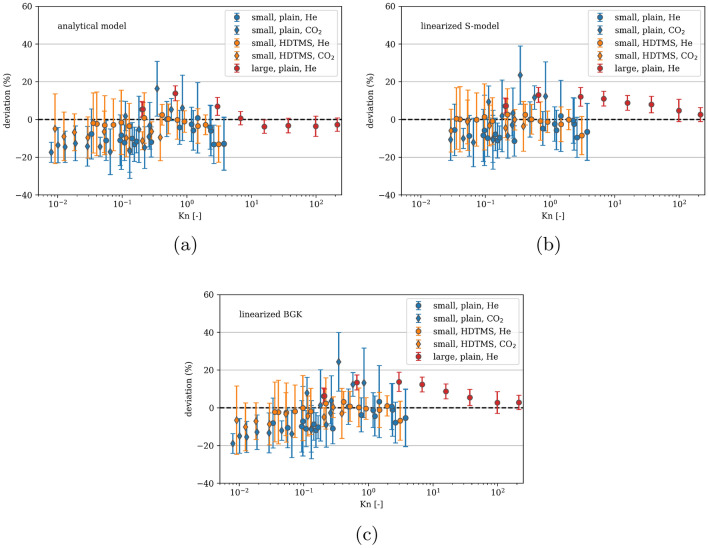
**(a)** Comparison of experiments to the ACM analytical model. A negative value corresponds to an experimental value smaller than the analytical one. **(b)** Comparison of experiments to the numerical solution of the linearized S-model equation^11^. **(c)** Comparison of experiments to the numerical solution of the linearized BGK equation.

The authors of^11^ have performed numerical simulations of flow through channels of rectangular cross-sections with different aspect ratios using the linearized S-model kinetic equation. The channel cross-section aspect ratio used in experiments made at the IUSTI is equal to 0.0359. This value is very close to the aspect ratio 0.0367 for which the simulations have already been done by the authors of^11^. Therefore, the data for dimensionless mass flow rate from Table 1 of Ref.^11^ is used to calculate the deviation of the experimental data from the numerical data and is plotted in Fig. 16b in Appendix 7. Good agreement was obtained even for the polyatomic gas. A TMAC of 1 seems to describe the experimental data well for both the plain and the functionalized surfaces.

### Analysis-based hydraulic closure model (ACM)

The ACM developed previously by the authors^13^ is used to calculate the mass flow rate under experimental conditions. The model is based on extensions of existing expressions for the mass flow rate through channels of various cross-sections and is fully predictive once both free parameters, the TMAC and the molecular diameter, have been determined. The authors of^13^ fitted the model on available experimental data for circular cross-sections from literature and obtained a so-called transition molecular diameter assuming diffuse reflection of the molecules on the surface. This means that the tangential momentum accommodation coefficient TMAC, which defines the fraction of diffusely reflected particles out of the total number of particles reflected from a surface, is assumed to be equal to 1 for circular cross-sections. For rectangular channels, the TMAC is set to 0.9 only for the analytical expression of the slip flow. In this work the model is applied as described in^13^ without any modification or adaption to the experimental data acquired resulting in good agreement between measured and calculated values of the mass flow rate, see Fig. 7c.

While the model uses a TMAC of 0.9 for the slip flow in rectangular channels, in^13^ it is stated that the actual TMAC should be 1 for all Knudsen numbers and the TMAC of 0.9 is attributed to a supposedly flawed slip expression for rectangular channels. The slip expression is derived from the solution of the Navier-Stokes equations using the Maxwell slip boundary condition and, for rectangular geometries, involves terms with convergent infinite series, while the expression for circular geometries is much simpler. There seems to be a complicating influence of the more complex rectangular geometry. A strong evidence that this is indeed the case is that if the TMAC was originally 0.9 for plain channels, it should become 1 when HDTMS is applied to the channel surfaces. HDTMS would sterically prevent any specular reflection, for which an even surface is needed^34^. Because the mass flow likely does not change to a large extent with functionalization, however, this means that the TMAC is 1 even for the plain channels. Both the calculations of the linearized S-model and BGK equations use a TMAC of 1, further strengthening this claim because of the good agreement with the experimental data.

## Conclusions

The method developed to make optimal use of the inherently noisy mass flow rate data is suitable to yield results with reasonable uncertainties. While the channels used here have a surface-to-volume ratio of 0.4 $\upmu$m^-1^, the mentioned mesopores have a ratio of around 300 $\mu$m^-1^ with a pore diameter of 20 nm. Here, the surface functionalization does not impact the mass flow rate through the channels considerably even though these experiments cover Knudsen numbers comparable to the experiments in mesoporous structures. This indicates that the Knudsen number is not the only parameter to consider but that there is a strong scaling dependence. The surface-to-volume ratio of the current channels (0.4 $\upmu$m^-1^) is too small for the surface functionalization to influence the mass flow rate in a way that clearly exceeds the measurement uncertainty. Probably the surface roughness which have not been measured here could decrease the impact of functionalization on the mass flow rate.

The measured mass flow rate is compared to numerical solutions of the linearized BGK and S-model kinetic equations and with an analysis-based hydraulic closure model (ACM) showing good agreement in all cases. The fully diffusive gas-surface interaction seems to describe both types of channel surfaces well.

The raw data of the small channels (plain and functionalized) have the following columns:

- X value: Time in seconds
- $T_1$: Room temperature measured on external wall of tank 1 in ^∘^C
- $T_2$: Room temperature measured on external wall of tank 1 in ^∘^C
- $V_{in}$: voltage of upstream pressure
- $V_{out}$: voltage of downstream pressure

To convert the voltages to pressure, following expression is used:

14 $$\begin{aligned} p_{in/out} = V_{in/out}\times \text{ sensor } \text{ range } \text{ in } \text{ Torr }/10 \times 133.32 \end{aligned}$$

For the raw data of TRANSFLOW experiments the header is explicitly given in the raw files.

## Figures and Tables

**Figure 9:**
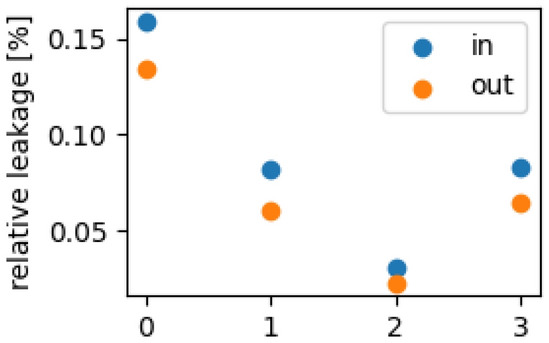
Relative leakage of |D2_CO2_1000_100_leakage_07-12-21|.

**Figure 10:**
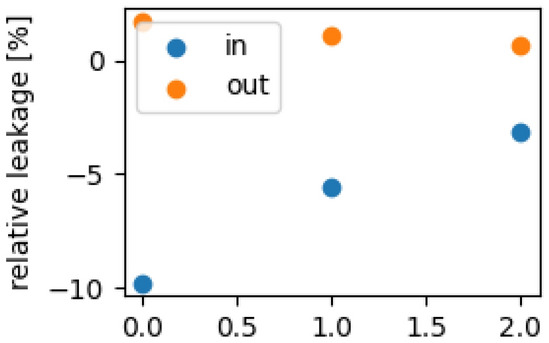
Relative leakage of |D2_He_1000_100_leakage_10k_14-01-22|.

**Figure 11:**
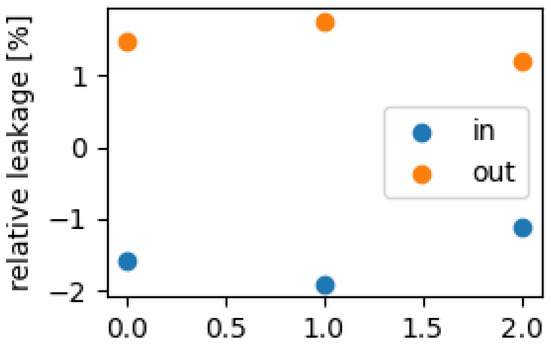
Relative leakage of |D2_He_100_100_leakage_1700_18-01-22|.

**Figure 12:**
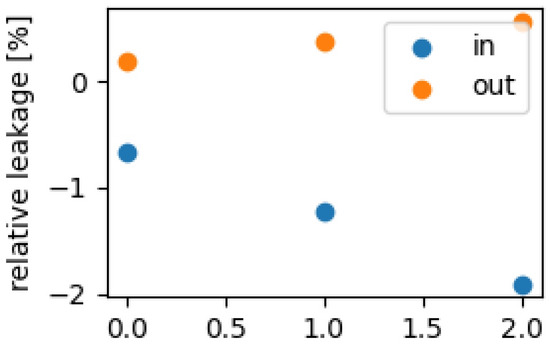
Relative leakage of |D2_He_1000_1000_leakage_42k_08-02-22|.

**Figure 13:**
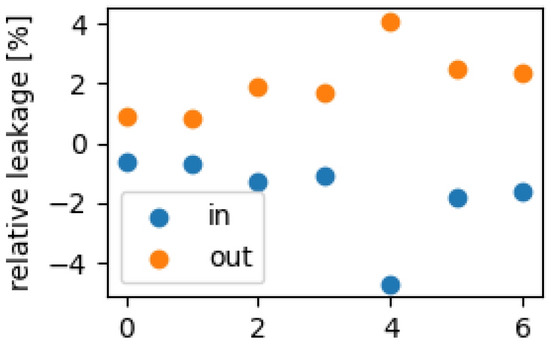
Relative leakage of |C2_He_100_100_leakage_700_18-03-22|.

**Figure 14:**
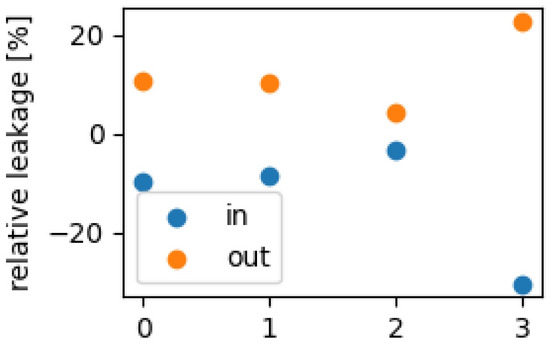
Relative leakage of |C2_CO2_100_100_leakage_14-03-22|.

**Figure 15:**
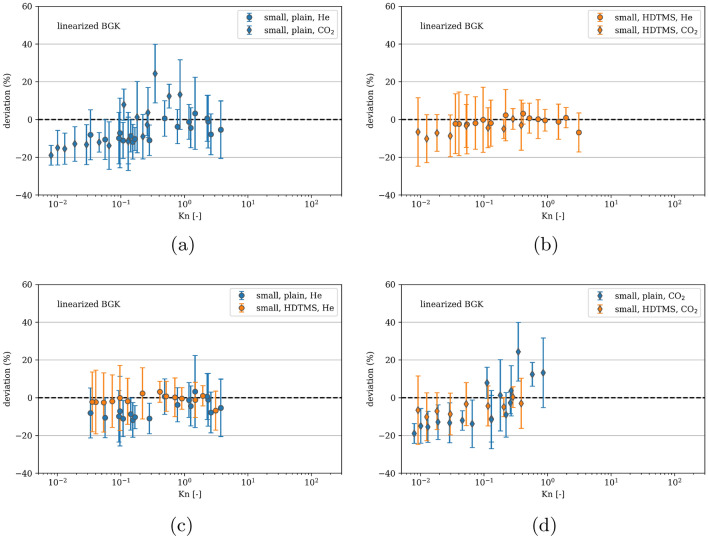
Deviations of experimental data from the solution of the linearized BGK equation. **(a)** Helium and carbon dioxide in plain channels. **(b)** Helium and carbon dioxide in functionalized channels. **(c)** Helium in plain and functionalized channels. **(d)** Carbon dioxide in plain and functionalized channels.

## Data Availability

The raw data are uploaded to Zenodo and they are accessible using the URL https://zenodo.org/records/10010163 or the DOI 10.5281/zenodo.10010162.

## Appendix A: Leakages

In this section the experimental data on the leakages are provided as well as the mass flow rates, for which the leak’ correction were used. The leakages have been measured in the inlet and outlet reservoirs using the sensors with the same or different full scales for two gases helium and carbon dioxide. First, the same pressure is set in the system, then the pressure variation in time in each tank is recorded. These measurements have been done for two configurations of the setup: with plain and functionalized channels. In total, six values of measured leakages are provided in the text given below. The corresponding files are accessible at Zenodo, see Data availability section above.

- type of channel: C2—functionalyzed, D2—plain
- type of a gas: CO_2_ or He
- type of sensor used to measure the pressure in inlet tank (inlet pressure) (full scale): 1000 (Torr), or 100 (Torr), or 10 (Torr)
- type of sensor used to measure the pressure in outlet tank (outlet pressure) (full scale): 1000 (Torr), or 100 (Torr), or 10 (Torr)
- value of the inlet and outlet pressure at the beginning of leak measurements (Pa)
- date of measurements

In the list of leakage files given below the value of the inlet/outlet pressure at the start of the experiment is missing for two files, namely:

- |D2_CO2_1000_100_leakage_07-12-21| the value of inlet/outlet pressure is 1000 Pa
- |C2_CO2_100_100_leakage_14-03-22| the value of inlet/outlet pressure is 50 Pa

The measured leaks make it possible to correct the mass flow values. Below, for each file containing leak information, the list of files with experimental conditions for mass flow measurements is given. These mass flow rates are corrected by the measured leaks. The relative effect of mass flow correction by leakage flow is given in the corresponding figures.

The structure of the name of a file with mass flow rate information is following

- type of channel: C2—functionalyzed, D2—plain
- type of a gas: CO_2_ or He
- type of sensor used to measure the pressure in inlet tank (inlet pressure) (full scale): 1000 (Torr), or 100 (Torr), or 10 (Torr)
- type of sensor used to measure the pressure in outlet tank (outlet pressure) (full scale): 1000 (Torr), or 100 (Torr), or 10 (Torr)
- value of the inlet pressure at the beginning of mass flow rate measurements (Pa) or (kPa)
- value of the outlet pressure at the beginning of mass flow rate measurements (Pa) or (kPa)
- date of measurements

1. |D2_CO2_1000_100_leakage_07-12-21|
  - inlet sensor: 1000
  - outlet sensor: 100
  - $${\dot{p}}_{1}$$: $$-5.915$$e−5 Pa/s
  - $${\dot{p}}_{2}$$: 3.561e−5 Pa/s
  - used by:
    - |D2_CO2_1000_100_massflow_5k_1k_08-12-21|
    - |D2_CO2_1000_100_massflow_11k_2k_09-12-21|
    - |D2_CO2_1000_100_massflow_22k_4k_09-12-21|
    - |D2_CO2_1000_100_massflow2_11k_2k_09-12-21| (Fig. 9)
2. |D2_He_1000_100_leakage_10k_14-01-22|
  - inlet sensor: 1000
  - outlet sensor: 100
  - $${\dot{p}}_{1}$$: 0.0355 Pa/s
  - $${\dot{p}}_{2}$$: 0.00664 Pa/s
  - used by:
    - |D2_He_1000_100_massflow_32k_12k_14-01-22|
    - |D2_He_1000_100_massflow_40k_11k_14-01-22|
    - |D2_He_1000_100_massflow_58k_11k_14-01-22| (Fig. 10)
3. |D2_He_100_100_leakage_1700_18-01-22|
  - inlet sensor: 100
  - outlet sensor: 100
  - $${\dot{p}}_{1}$$: 0.000945 Pa/s
  - $${\dot{p}}_{2}$$: 0.000973 Pa/s
  - used by:
    - |D2_He_100_100_massflow_5200_1100_18-01-22|
    - |D2_He_100_100_massflow_6k_3k_18-01-22|
    - |D2_He_100_100_massflow_10k_5k_18-01-22|
4. |D2_He_1000_1000_leakage_42k_08-02-22|
  - inlet sensor: 1000
  - outlet sensor: 1000
  - $${\dot{p}}_{1}$$: 0.0129 Pa/s
  - $${\dot{p}}_{2}$$: 0.00485 Pa/s (Fig. 11)
  - used by:
    - |D2_He_1000_1000_massflow_133k_90k_08-02-22|
    - |D2_He_1000_1000_massflow_83k_49k_08-02-22|
    - |D2_He_1000_1000_massflow_56k_25k_08-02-22| ((Fig. 12))
5. |C2_He_100_100_leakage_700_18-03-22|
  - inlet sensor: 100
  - outlet sensor: 100
  - $${\dot{p}}_{1}$$: 0.000861 Pa/s
  - $${\dot{p}}_{2}$$: 0.00115 Pa/s
  - used by:
    - |C2_He_100_100_massflow_12k_3k_18-03-22|
    - |C2_He_100_100_massflow_13k_5k_18-03-22|
    - |C2_He_100_100_massflow_6500_1600_18-03-22|
    - |C2_He_100_100_massflow_7900_2600_18-03-22|
    - |C2_He_100_100_massflow_1950_400_18-03-22|
    - |C2_He_100_100_massflow_3400_430_18-03-22|
    - |C2_He_100_100_massflow_4300_700_18-03-22| (Fig. 13)
6. |C2_CO2_100_100_leakage_14-03-22|
  - inlet sensor: 100
  - outlet sensor: 100
  - $${\dot{p}}_{1}$$: 0.00192 Pa/s
  - $${\dot{p}}_{2}$$: 0.00217 Pa/s
  - used by:
    - |C2_CO2_100_100_massflow_5k_1k_14-03-22|
    - |C2_CO2_100_100_massflow_6k_2k_14-03-22|
    - |C2_CO2_100_100_massflow_12k_3k_14-03-22|
    - |C2_CO2_100_100_massflow_12k_3k_14-03-22| (Fig. 14)

## Appendix B: Deviation of experimental data from linearized BGK model

Figure 15 visualizes the relative deviation of the experimental data from the linearized BGK model.

## Appendix C: Deviations of experimental data from models

Figure 16 visualizes the relative deviation of the experimental data from each baseline. This figure reproduce one part of Fig. 15, namely (c) and (d), but the results with S-model are added to make up an ensemble of figures which are best viewed side-by-side.
